# Generation of osteoclast-like cells from human peripheral blood mononuclear cells using *NFATc1* modified RNA

**DOI:** 10.1371/journal.pone.0342642

**Published:** 2026-02-11

**Authors:** Sirada Srihirun, Chareerut Phruksaniyom, Thanaporn Sriwantana, Tipparat Parakaw, Nathawut Sibmooh, Pornpun Vivithanaporn, Kran Suknuntha

**Affiliations:** 1 Department of Pharmacology, Faculty of Dentistry, Mahidol University, Bangkok, Thailand; 2 Chakri Naruebodindra Medical Institute, Faculty of Medicine Ramathibodi Hospital, Mahidol University, Samut Prakan, Thailand; Northwest Institute of Plateau Biology Chinese Academy of Sciences, CHINA

## Abstract

Inhibition of osteoclasts, a type of bone-resorptive cell, is a key approach for treating diseases associated with bone loss. Although human osteoclasts can be generated from receptor activator of nuclear factor-ĸB ligand-mediated stimulation of peripheral blood mononuclear cells (PBMCs) in vitro, the differentiation rate is low and the process is time consuming. Here, we aimed to generate osteoclast-like cells from human PBMCs using modified RNA (modRNA) enforcing *NFATc1*, a master transcription factor that functions downstream of receptor activator of nuclear factor-ĸB receptor activation. Human PBMCs isolated from the venous blood of healthy donors (n = 3) using Histopaque density gradient centrifugation were expanded in medium supplemented with 25 ng/mL macrophage colony-stimulating factor for 3 days. The isolated macrophages were transfected with *NFATc1* modRNA. The osteoclast-like cells were evaluated for the following osteoclast-specific markers: tartrate-resistant acid phosphatase-positive expression, F-actin ring formation, cathepsin expression, and bone resorption activity. Scanning electron microscopy was used to morphologically characterize the osteoclast-like cells. At day 1 posttransfection with 25, 50, and 100 ng/mL of modRNA, *NFATc1* expression in human macrophages increased by 1.20 ± 0.08-fold, 3.47 ± 1.02-fold, and 13.31 ± 3.96-fold, respectively. Transfection with 25 or 50 ng/mL of *NFATc1* modRNA for 3 days induced the formation of tartrate-resistant acid phosphatase-positive multinucleated cells, the F-actin ring formation, expression of cathepsin K, and increased bone resorption activity. Scanning electron micrographs confirmed the presence of round, multinucleated cells with projecting microvilli, characteristic of osteoclasts. In conclusion, rapid generation of functional osteoclast-like cells was achieved in vitro using *NFATc1* modRNA. This method offers faster results compared with conventional receptor activator of nuclear factor-ĸB ligand stimulation.

## Introduction

Periodontitis, one of the most common inflammatory diseases of the oral cavity, can become severe and lead to tooth loss owing to insufficient alveolar bone support. Osteoclasts are key players in many osteolytic diseases; for example, increased osteoclast activity is associated with pathological bone loss in osteoporosis and periodontitis [1,2]. Osteoclasts have been shown to be stimulated following secretion of the bacterial lipopolysaccharide (LPS)-induced receptor activator of nuclear factor-ĸB ligand (RANKL) [3,4]. Therefore, modulation of excessive osteoclast activity may prevent adverse outcomes in periodontal diseases. Although osteoclasts are primarily derived from hematopoietic stem cells in the bone marrow, in vitro-differentiated osteoclasts are routinely used as model cells for the investigation of new anti-resorptive drugs. Osteoclasts are generated in vitro by stimulating primary, bone marrow-derived cells or murine cell lines with RANKL [5]. Disadvantages of this approach include the variation in differentiation rate, lengthy time requirement, short lifespan of progenitor cells, and genetic manipulation of osteoclast precursors [6,7].

NFATc1, a transcription factor acting downstream of activated RANKL, plays an important role in osteoclast differentiation [8]. NFATc1 regulates osteoclast differentiation by inducing the expression of osteoclast-specific genes encoding tartrate-resistance acid phosphatase (TRAP), cathepsin K, calcitonin receptor, matrix metalloproteinase-9, and vacuolar-type ATPase. In one study, the ectopic expression of NFATc1 induced RANKL-dependent osteoclast formation in bone marrow-derived macrophages, while cells with NFATc1 knockout failed to differentiate to osteoclasts [9].

Modified RNA (modRNA) is new gene delivery technique that is widely used to induce cell differentiation. However, for difficult-to-transfect cells (primary, immune, and stem cells), gene delivery by retrovirus or lentivirus demonstrates superior transfection efficiency [10]. Given the risk of mutagenesis of viral vectors that randomly integrate into the host genome [11], synthetic modRNA has become an interesting alternative tool for the induction of transient protein expression in target cells. Proteins of interest encoded by modRNA are synthesized in the cytoplasm (no nuclear translocation required), enabling fast, localized expression [12,13]. This study aimed to generate an osteoclast model from human primary mononuclear cells using *NFATc1* modRNA. The *NFATc1*-induced osteoclast-like cells were then examined for morphology, specific osteoclast biomarkers, and functional activities.

## Methods

### Ethical statement

This research was approved by the Ethical Committee of the Faculty of Dentistry/Faculty of Pharmacy, Mahidol University (COA 2022/022.1705), in accordance with the principles of the Declaration of Helsinki. The subjected were recruited during 17 May 2022 − 17 May 2024. Human venous blood was isolated from female healthy participants (n = 3), who provided written informed consent before enrolling in this study.

### Construction of in vitro transcription (IVT) templates and synthesis of modRNA

*NFATc1* (GeneID 4772) and eGFP IVT templates were generated using the pGEM T easy vector and 120-A tract primers, as described previously [14]. *NFATc1* template was generated from U937-derived osteoclasts using conventional phorbol 12-myristate 13-acetate (PMA) and 1α, 25 di-hydroxy Vitamin D_3_. Subsequent PCR identified the isoform J (NM_001278673.2) of *NFATc1* was isolated. ModRNA was synthesized with the HiScribe™ T7 High-yield RNA synthesis kit (New England Biolabs, Ipswich, MA, USA), using a custom ribonucleoside cocktail comprising CleanCap^®^ Reagent AG (TriLink Biotechnologies, San Diego, CA, USA), pseudouridine triphosphate, adenosine triphosphate, guanosine triphosphate, and cytidine triphosphate, in accordance with the manufacturer’s instructions. Reactions were incubated for 3 hours at 37 °C and then treated with DNase. The RNA was purified using RNA Clean & Concentrator (Zymo Research, Irvine, CA, USA) and adjusted to 100 ng/μL with RNase-free water before freezing at ˗80 °C.

### Isolation of human peripheral blood mononuclear cells (PBMCs) and cell culture

PBMCs were isolated from approximately 50 mL of fresh venous blood collected from healthy volunteers using density gradient centrifugation with Histopaque-1077 (Sigma-Aldrich, St. Louis, MO, USA), in accordance with the manufacturer’s protocol. Centrifugation was performed at 400g for 30 minutes, without a break, at room temperature. The layers containing PBMCs were collected and washed once with phosphate-buffered saline (PBS, pH 7.4). The PBMCs were resuspended in freezing medium and stored in liquid nitrogen until use.

Human macrophages were isolated from PBMCs after expansion in completed Dulbecco’s modified Eagle’s medium (DMEM) supplemented with 10% fetal bovine serum and 1% penicillin/streptomycin, in the presence of 25 ng/mL human macrophage colony-stimulating factor (M-CSF; R&D Systems, Minneapolis, MN, USA). After a 3-day expansion, macrophages were detached using a cell scraper and seeded (1.5 × 10^5^ cells) in 48 well-plates or 8-well chamber slides (Ibidi, Fitchburg, WI, USA) or 1.5 x 10^6^ cells in 6 well-plate.

### modRNA transfection

Macrophages were expanded in complete medium supplemented with 25 ng/mL M-CSF until cells reached 80% confluence (2–4 days), and then cultured for another 2 days without M-CSF prior to transfection. *NFATc1* modRNA or eGFP modRNA (25−100 ng/mL) was transfected into human macrophages using MessengerMAX Lipofectamine (Thermo Fisher Scientific, Waltham, MA, USA), in accordance with the manufacturer’s protocol in 48-well plate. The transfection efficiency of *NFATc1* modRNA was evaluated at 24 hours posttransfection using real-time PCR and as follows. Total RNA was extracted and purified using an RNA purification kit (Favorgen Biotech Corp., Ping Tung, Taiwan). The cDNA was synthesized using an iScript cDNA synthesis kit (Bio-Rad, Hercules, CA, USA). Quantitative real-time PCR was conducted using a KAPA SYBR FAST kit (KAPA Biosystem, Wilmington, MA, USA) and self-designed primers specific for *CTSK*, *ATP6V0A1*, *MMP-9, ITGB3,* and *CAII* on a Real-Time PCR CFX96 (Bio-Rad). Gene expression values were calculated by subtracting the Ct value of the reference gene *GAPDH* from that of the target gene, and expressed as relative fold change (2-^ΔΔCT^method). Undifferentiated cells were used for calibration.

### RANKL-induced osteoclast differentiation

Human macrophages were plated (1.5 × 10^**5**^ cells) in 48 well-plates with 25 ng/mL M-CSF and cultured overnight at 37 °C in CO_**2**_ incubator. For osteoclast differentiation, 50 ng/mL RANKL was added to the cultures with or without M-CSF for 7 days. Osteoclast formation was evaluated by TRAP staining assay.

### Western blot

Twenty-four hours after *NFATc1* modRNA transfection, cells were washed with PBS and lysed using lysis buffer (50 mM Tris, 150 mM NaCl, and 0.5% NP-40) with proteinase inhibitor cocktail III (1:500, Calbiochem, La Jolla, CA, USA). Protein was loaded on 8% polyacrylamide gel and transferred to the nitrocellulose membrane. The membranes were blocked with 5% skimmed milk for 1 hour and subsequently incubated with primary antibodies, including NFATc1 (Cell Signaling, #8032) and β-actin (Cell Signaling, #4970) at 4 °C overnight. The horseradish peroxidase-conjugated secondary antibodies were incubated for 2 hours and followed by enhanced chemiluminescence (ECL) detection (Bio-Rad). Band density was quantified by Amersham Imagequant 800 (GE Healthcare, Piscataway, NJ, USA).

### Cell viability assay

The cell viability was evaluated using calcein-AM/propidium iodide staining assay. At day 3 posttransfection, the cells were gently washed with PBS and stained with calcein-AM (Invitrogen, #C34852) and propidium iodide (Invitrogen, #P1304MP) according to the manufacturing protocol for 30 minutes at 37 °C. The fluorescence imaging was captured by an inverted microscope (Olympus CKX53, Tokyo, Japan).

### TRAP staining

TRAP staining was performed on day 3 posttransfection with *NFATc1* modRNA using a TRAP staining kit (Cosmo Bio, Carlsbad, CA, USA). Cells were fixed with a 10% neutral-buffered formalin solution (30 mM NaH_2_PO_4_ and 45 mM Na_2_HPO_4_) at room temperature for 5 minutes. After TRAP staining, images were captured by 10X objective lens CKX53 inverted (Olympus Corporation, Tokyo, Japan). Cells containing at least 3 nuclei with positive stain were counted as osteoclasts.

### Immunofluorescence staining

Immunofluorescence staining was performed for actin rings on days 1, 2, and 3 and for cathepsin K on day 3 *NFATc1* modRNA posttransfection. Cells were fixed with 4% paraformaldehyde at room temperature for 10 minutes, permeabilized with 0.1% Triton X-100 for 5 minutes, blocked with 5% bovine serum albumin for 30 minutes. Cells were stained with 1:100 anti-cathepsin K (Santacruz, #sc-376803,) at 4 °C overnight and further stained with 1:1000 Rhodamine anti-mouse secondary (Invitrogen, #31660) antibody for 1 hour. F-actin ring was stained with 1:1000 iFluoro488-phalloidin (Abcam,#ab176753) for 1 hour and mouthed with prolong gold anti-fade DAPI to nuclei staining (Cell signaling,#8961). Images were randomly taken using the spiral scan path of the confocal microscope (Stellaris 5, Leica Microsystems, Wetzlar, Germany).

### Scanning electron microscopy (SEM)

Macrophages were seeded at a density of 1.5 × 10^5^ cells on surface-modified coverslips and transfected with *NFATc1* modRNA for 3 days. The cells were then sequentially fixed with 2.5% glutaraldehyde at 4 °C for 2 hours and 1% OsO_4_ at room temperature for 2 hours. Specimens were dehydrated through a graded ethanol series and air dried. The surface of the samples was coated with gold ion sputter and photographed with a scanning electron microscope (JEOL, Tokyo, Japan).

### Bone resorption assay

Macrophages were seeded at a density of 1.5 × 10^5^ cells in a fluoresceinamine-labeled, calcium phosphate-coated 48-well plate (Cosmo Bio, Carlsbad, CA, USA) in DMEM without phenol red in the presence of 25 ng/mL M-CSF. Three days after *NFATc1* modRNA transfection, the fluoresceinamine-labeled chondroitin sulfate (FACS) containing conditioned medium from each well was transferred into a black 96-well plate for fluorescence measurements at 485 and 535 nm. Cells were removed by treatment with 5% sodium hypochlorite for 5 min. The bottom of the well was photographed by an Olympus CKX53 inverted microscope equipped with an Olympus DP22 color camera.

### Statistical analysis

Data are presented as means ± standard error of the mean. Data processing and statistical analysis were performed using Prism^®^ software version 6 (GraphPad Software Inc., San Diego, CA, USA). Statistical differences, defined as a *p* value < 0.05, were evaluated using either Student’s t-test or ANOVA with Tukey’s multiple comparison test where indicated. Significant levels were determined compared to control or lipofectamine as indicated

## Results

### Dose-dependent expression of transfected *NFATc1* modRNA in macrophages

**Fig 1:**
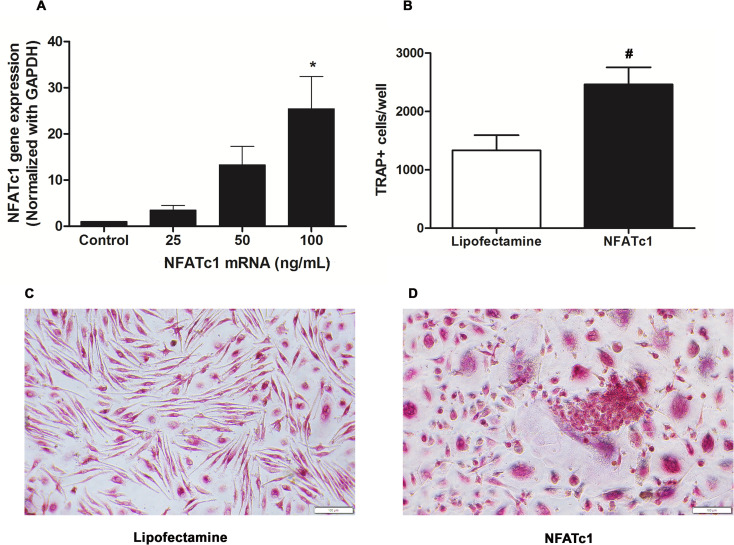
TRAP^Bright^ multinucleated cells induced by transfection of *NFATc1* modRNA. **(A)** Expression of *NFATc1* modRNA in human macrophages at 24 hours posttransfection. Data are mean ± SEM (n = 3). * *p* < 0.05 tested by ANOVA with Tukey’s multiple comparison. **(B)** Numbers of TRAP^Bright^ cells following treatment with Lipofectamine (control) or transfection with 50 ng/mL of *NFATc1* modRNA (n = 3). Data are mean ± SEM (n = 3). # *p* < 0.05 tested by t-test **(C, D)** Representative images of the formalin-fixed, TRAP^Bright^ cells described in (B). Scale bars = 100 µm.

eGFP modRNA was used as an internal control to evaluate for RNA quality and tranfection/translation efficiency of the synthetic modRNA. Twenty-four hours after eGFP modRNA transfection, bright green fluorescent acitivity can be observed in the macrophages (S1 Fig). Similary, a 24-hour transfection with *NFATc1* modRNA (25, 50, and 100 ng/mL) into wells containing 80% confluent macrophages increased *NFATc1* RNA levels by 1.20 ± 0.08-fold, 3.47 ± 1.02-fold, and 13.31 ± 3.96-fold compared with the control, respectively (Fig 1A). A time-course analysis showed that the downstream *NFATc1* target genes crucial for osteoclast differentiation reached peak levels 24 hours after transfection (S2 Fig). The protein expression of NFATc1 was confirmed by western blot (S3 Fig). On day 3 posttransfection, significant cytotoxicity was observed at 100 ng/mL (S4 Fig). Adherent macrophages became round and eventually detached from the surface of the well. Therefore, *NFATc1* modRNA was used at 25 and 50 ng/mL for subsequent experiments.

### Emergence of TRAP^Bright^ multinucleated cells after forced *NFATc1* expression

Starting on day 2 posttransfection with 50 ng/mL *NFATc1* modRNA, the macrophages changed from fusiform to round-shaped cells and began exhibiting multinucleation. The multinucleated cells, which gradually emerged during culture and increased in number from 1,335 ± 260–2,463 ± 292 cells/well after day 3 (Fig 1B). TRAP is a metalloprotein enzyme expressed by osteoclasts, activated macrophages, and neurons. Thus, evaluation using multiple features including multinucleation, TRAP expression, and round-shaped morphology were required to diffenretiate osteoclasts from the background macrophages. We found that the emerging multinucleated cells were positive for TRAP (TRAP^Bright^) and show all of the above features of osteoclasts (Fig 1C and 1D). In contrast, RANKL significant induced TRAP^Bright^ multinucleated cells at day 7 (S5 Fig).

### *NFATc1* modRNA-induced expression of F-actin and cathepsin K

**Fig 2:**
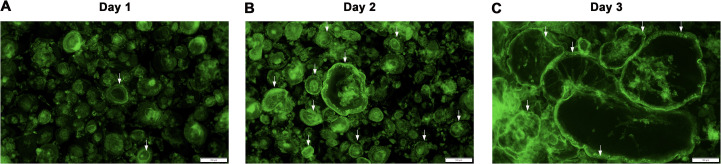
*NFATc1* modRNA-induced F-actin ring formation. Representative fluorescence microscopy images of F-actin formation in human macrophages on day 1 **(A)**, 2 **(B)** and 3 **(C)** posttransfection of *NFATc1* modRNA. *NFATc1* modRNA at 50 ng/mL was transfected in human macrophages and stained with iFluoro488-conjugated phalloidin (green) in human macrophages (n = 3). Scale bars = 50 µm.

**Fig 3:**
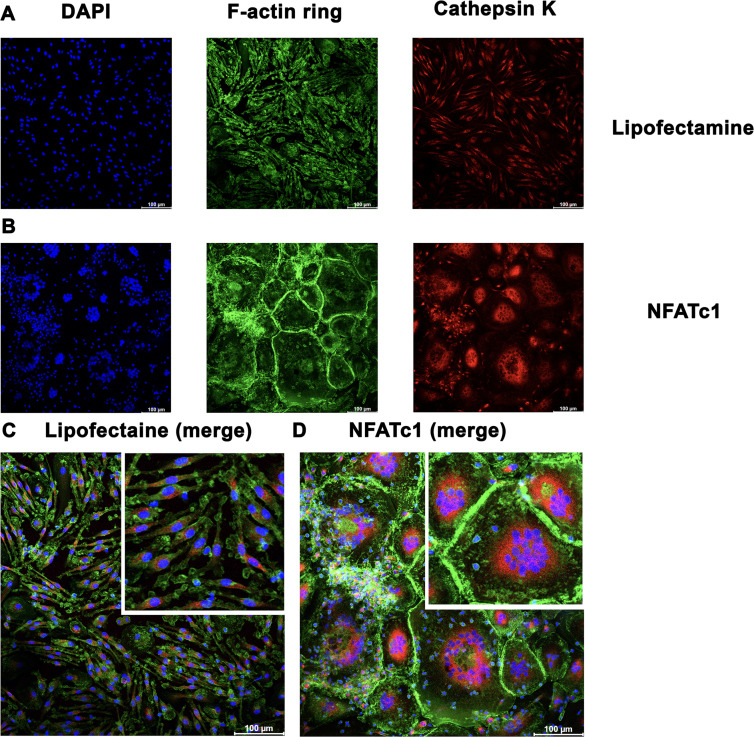
F-actin and cathepsin K expression following transfection of *NFATc1* modRNA. Representative confocal microscopy images showing upregulated F-actin formation and cathepsin K expression in human macrophages at 3 days posttransfection of 50 ng/mL *NFATc1* modRNA.

The F-actin ring or sealing zone is a critically important, characteristic structure of osteoclasts that forms during bone resorption [15]. We observed podosome clusters of F-actin as early as 24 hours posttransfection, while podosome belts and podosome rings developed later, at 48–72 hours posttransfection (Fig 2). Cathepsin K is an essential bone-resorptive enzyme secreted by osteoclasts located in the resorptive lacuna [16]. At 72 hours posttransfection, *NFATc1* modRNA also induced the expression of cathepsin K, which was localized at the center of the cells (Fig 3).

Cells were fixed and permeabilized with 0.1% Triton X-100 for 5 minutes, and stained for F-actin rings with iFluoro488-conjugated phalloidin (green), cathepsin K with rhodamine (red), and nuclei with DAPI (blue) (n = 3). Scale bars = 100 µm.

### Bone-resorption activity triggered by *NFATc1* modRNA

**Fig 4:**
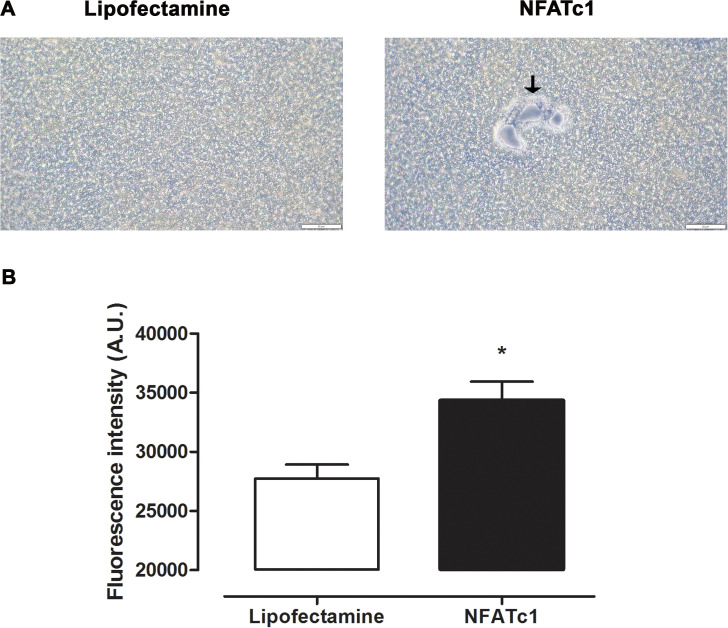
Bone resorption activity of *NFATc1* modRNA-induced osteoclasts. **(A)** Representative images of resorptive pits (empty spaces around multinucleated cells; arrows) in human macrophages transfected with Lipofectamine (control) or 50 ng/mL *NFATc1* modRNA and cultured on a FACS calcium phosphate-coated plate for 3 days. Scale bars = 50 µm. **(B)** Free calcium phosphate levels in the culture medium of (A); * *p* < 0.05, analyzed by t-test (n = 3).

Although multinucleation, morphology, and certain biomarkers are supportive of the osteoclast phenotype, the most crucial feature is bone-resorption activity [17]. To simultaneously visualize and quantify the bone-resorption activity of *NFATc1*-induced osteoclasts, we performed a pit-formation assay using FACS calcium phosphate-coated plates [18]. In this assay, the resorbed calcium phosphate is released in the medium as free ions, which can be quantified through the fluorescent activity. *NFATc1*-induced osteoclasts exhibited pit formation and the release of free calcium phosphate (Fig 4A and 4B).

### SEM of *NFATc1*-induced osteoclasts

**Fig 5:**
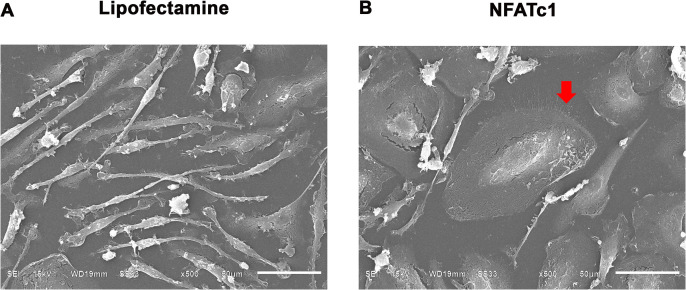
Scanning electron microscopy. SEM micrographs of Lipofectamine (control) or 50 ng/mL *NFATc1* modRNA-induced osteoclasts at day 3 posttransfection (n = 3). Red arrow indicates osteoclast with microvilli projections. Scale bars = 50 µm.

Under SEM, *NFATc1-*induced osteoclasts exhibited round morphology, multinucleation, and cytoplasmic microvilli projecting from the cell surface (Fig 5). In contrast, mock-transfected macrophages remained small, with spindle-shaped morphology and no cytoplasmic microvilli.

## Discussion

Periodontal inflammation is a key pathologic finding of periodontitis that is usually accompanied by osteoclastogenesis [2], resulting in the loss of alveolar bone support in the late stage of disease. Given the rarity of osteoclasts in the periodontal tissue, in vitro models are invaluable tools for the study of osteoclast biology in health and disease. As multinucleated cells derived from monocytes or macrophages [19], osteoclasts play major roles in the maintenance and remodeling of bone. The tumor necrosis-related cytokine RANKL and the polypeptide growth factor colony-stimulating factor-1 (CSF-1) are essential [20,21] for the activation of receptor activator of nuclear factor-ĸB on the surface of hematopoietic precursors [22,23]. A combination of cytokines has previously been used to direct osteoclast differentiation of monocyte-macrophage lineage cells [24] and PBMCs [25]. However, this is a lengthy approach, requiring 7 and 17 days to generate the first wave of osteoclasts in the mouse and human models, respectively. Therefore, an alternative method with rapid results is desired.

With the advent of new chemicals, synthetic RNA has become reliably stable and easy to handle. Indeed, the rapid onset of modRNA expression has made it a widely used method for transient gene delivery [12,13], leaving no residual footprint in the target cells. In the present study, we demonstrated the rapid production of osteoclast-like cells from PBMCs using *NFATc1* modRNA. The generated osteoclasts exhibited several osteoclast characteristics, including round shape, multinucleation, F-actin ring formation, and expression of TRAP and cathepsin K. The first wave of TRAP^bright^ multinucleated cells was observed as early as 3 days posttransfection, while the conventional method requires 17 days [25]. Importantly, these osteoclast-like cells possessed bone resorption ability, as defined by the demineralization of coated calcium phosphate and release of free calcium ions into culture medium. We noticed that *NFATc1* modRNA-induced osteoclasts demonstrate low resorption activity. We hypothesize that *NFATc1* alone maybe insufficient to induce optimal bone resorption activity or the modRNA-induced innate immune activation in the macrophages may inhibit their functionality [26,27]. B18R, a vaccinia virus-encoded protein, can reduce the innate immune response after mRNA transfection by acting as a decoy receptor binding to type I interferons (IFNs). Thus, addition of B18R may allow longer expression of the *NFATc1* mRNA and improved efficiency [28–30]. Although a pit formation assay on intact bone is the gold standard for assessing resorption ability, performing this assay remains a technical challenge in our setting. Furthermore, a very low amount of modRNA (≥ 25 ng/mL in a 48-well plate) was needed to successfully generate osteoclast-like cells. While raising the mod RNA to 100 ng/mL increased *NFATc1* RNA levels by up to 15 fold, it did not further enhance osteoclast differentiation, but rather increased cytotoxicity to the macrophages. Additionally, we observed that the optimal dose of modRNA (25–50 ng/mL) varied among the PBMCs derived from different donors, thus the balance between differentiation and cytotoxicity must be determined for each donor. Although the modRNA method is rapid and has higher efficiency in generating TRAP^bright^ cells than the cytokine method (S5 Fig), scaling up the experiment still requires further optimization. In contrast, the conventional cytokine method usually starts with a culture of large numbers of PBMCs and requires a longer culture period (i.e., 14 days), resulting in higher numbers of osteoclasts [25,31,32]. Considering that several transcription factors cooperate to control the fate of osteoclasts, transfection of a single modRNA-encoded transcription factor such as *NFATc1* is likely ineffective for mass production.

## Conclusion

Our study demonstrates that a single *NFATc1* modRNA is sufficient for direct osteoclast differentiation in PBMCs isolated from peripheral blood. We anticipate that a platform of in vitro generated osteoclasts will be a valuable tool for high-throughput drug screening and disease modeling, and may have potential in cell-based therapy. Further studies should aim to improve the differentiation efficiency and production scale of osteoclast-like cells.

## Supporting information

S1 Fig eGFP expression in human macrophages after 24-hour transfection at 100 ng/mL. Representative fluorescent image of human macrophages after transfection with eGFP modRNA for 24 hours.
(JPG)

S2 Fig Time-course analysis of *NFATc1* target genes by qPCR. Expression of *ITGB3*, *MMP-9*, *V-ATPase*, *CTSK*, and *CAII* genes was detected at 1–2 days post transfection with *NFATc1* modRNA by qPCR. Dash line indicated baseline expression of control. Data are mean ± SEM (n = 4). * *p* < 0.05 tested by ANOVA with Tukey’s multiple comparison.
(TIF)

S3 Fig *NFATc1* protein expression in human macrophages after *NFATc1*-modRNA transfection at 25, 50 and 100 ng/mL. Representative western blots of NFATc1 (A) and quantitative of band intensity of NFATc1/β-actin (B) in human macrophages after transfection with *NFATc1* modRNA for 24 hours. Data are mean ± SEM (n = 5). * *p* < 0.05 tested by ANOVA with Tukey’s multiple comparison.
(TIF)

S4 Fig Cell viability of human macrophages posttransfection with *NFATc1* modRNA. Human macrophages were transfected with 25, 50, 100 and 200 ng/mL *NFATc1* modRNA for 3 days (A-E). Cell viability was measured by calcein-AM/PI staining assay. Green and red color represent living and dead cells, respectively. The percentage of living cells after *NFATc1* transfection for 24 hours (F). Scale bar. 100 um. Data are mean ± SEM (n = 3). *p < 0.05 tested by ANOVA with Tukey’s multiple comparison.
(TIF)

S5 Fig TRAP^Bright^ multinucleated cells induced by RANKL. Representative images of TRAP^Bright^ cells from treated human macrophage with 25 ng/mL M-CSF (A, C) and 25 ng/mL M-CSF with 50 ng/mL RANKL (B, D) for 3 and 7 days. Scale bars = 200 µm. (E) Numbers of TRAP^Bright^ cells following treatment with M-CSF or M-CSF and RANKL for 7 days. Data are mean ± SEM (n = 3). * *p* < 0.05 tested by t-test.
(TIF)
