# Supplementary figures and images for: Generation of osteoclast-like cells from human peripheral blood mononuclear cells using NFATc1 modified RNA
Source: PLoS One. 2026 Feb 11;21(2):e0342642. doi: 10.1371/journal.pone.0342642 (PMC12893540; doi:10.1371/journal.pone.0342642)

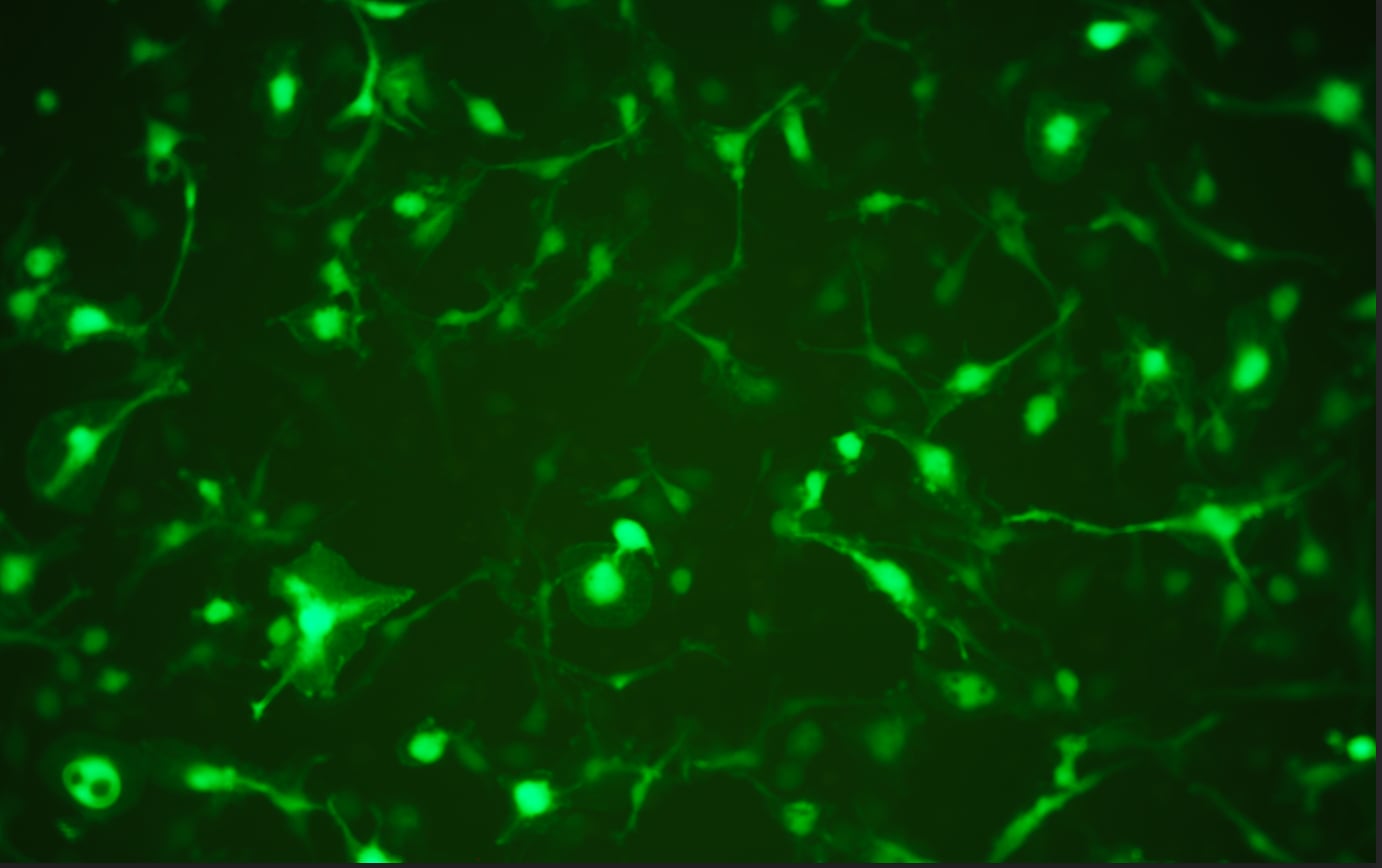

Supplement: S1 Fig — Representative fluorescent image of human macrophages after transfection with eGFP modRNA for 24 hours. (JPG) [file pone.0342642.s001.jpg]

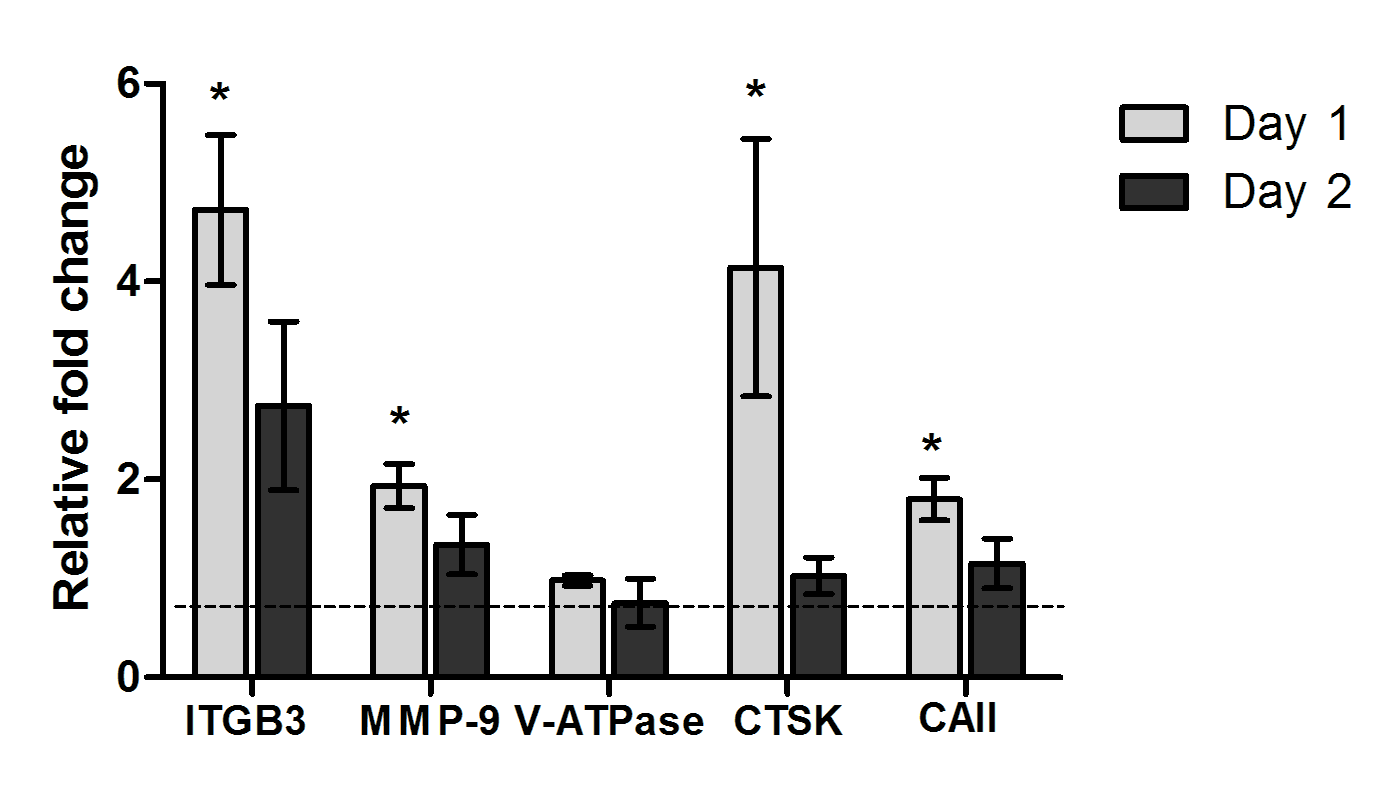

Supplement: S2 Fig — Expression of ITGB3, MMP-9, V-ATPase, CTSK, and CAII genes was detected at 1–2 days post transfection with NFATc1 modRNA by qPCR. Dash line indicated baseline expression of control. Data are mean ± SEM (n = 4). * p < 0.05 tested by ANOVA with Tukey’s multiple comparison. (TIF) [file pone.0342642.s002.tif]

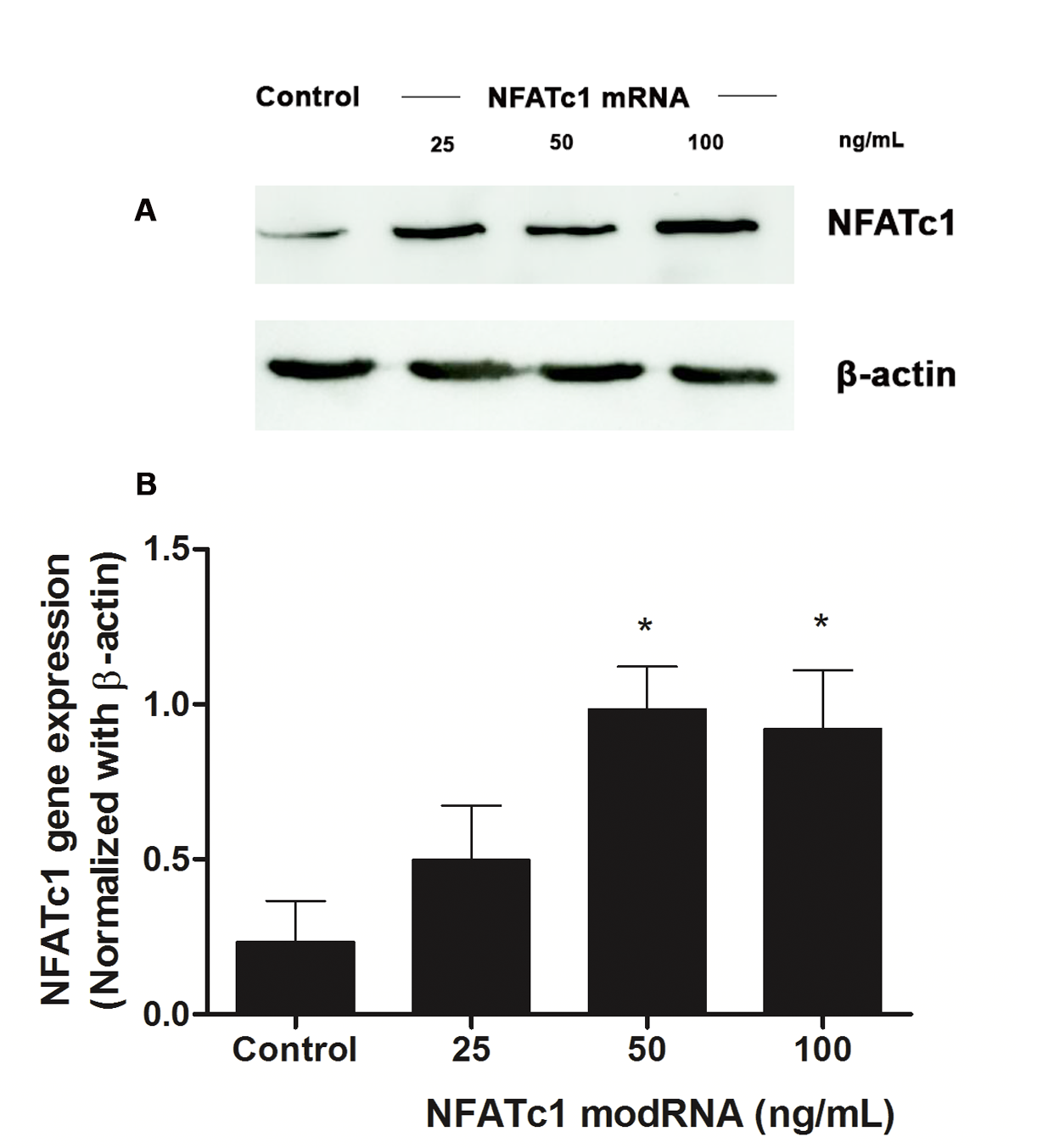

Supplement: S3 Fig — Representative western blots of NFATc1 (A) and quantitative of band intensity of NFATc1/β-actin (B) in human macrophages after transfection with NFATc1 modRNA for 24 hours. Data are mean ± SEM (n = 5). * p < 0.05 tested by ANOVA with Tukey’s multiple comparison. (TIF) [file pone.0342642.s003.tif]

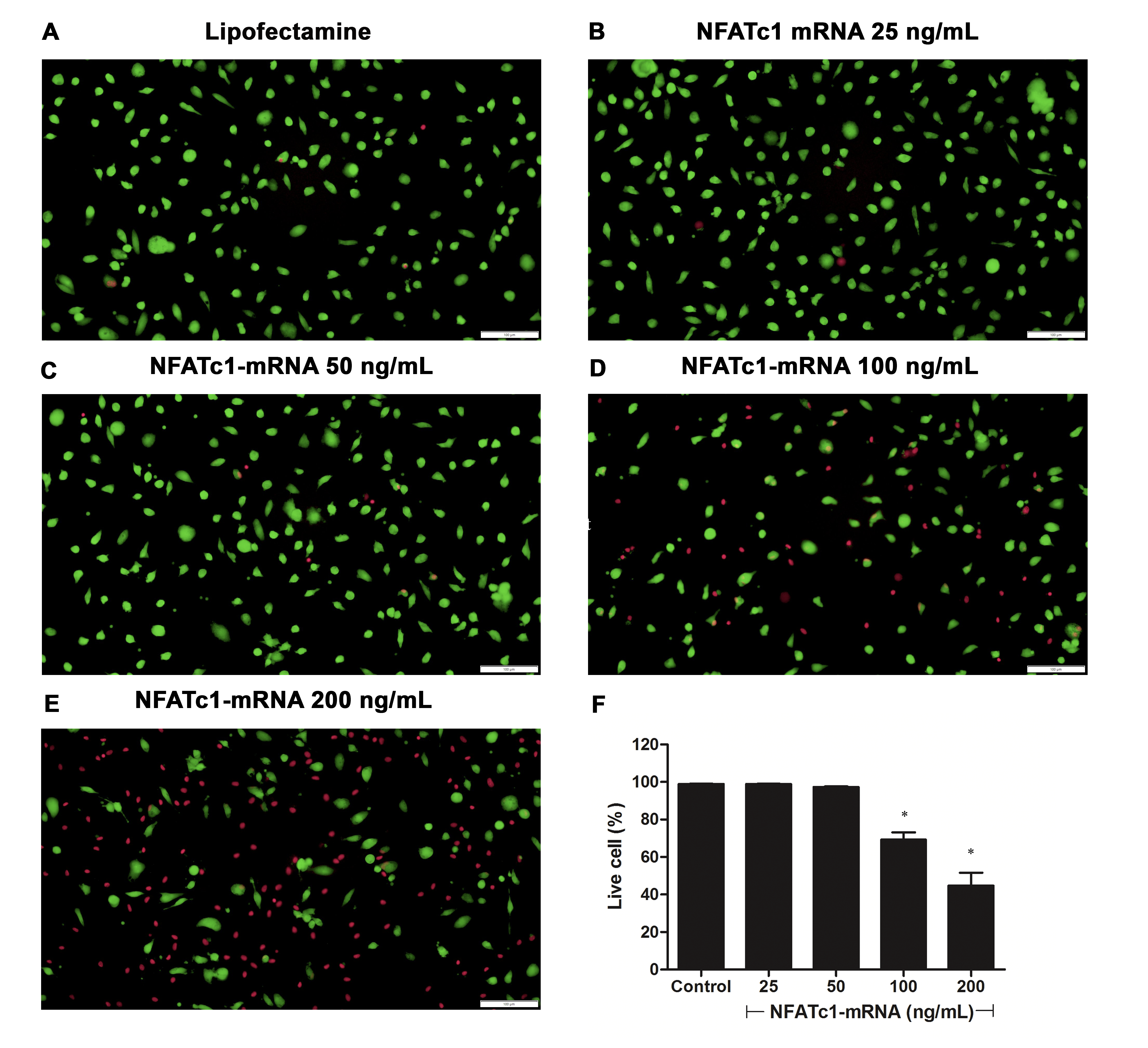

Supplement: S4 Fig — Human macrophages were transfected with 25, 50, 100 and 200 ng/mL NFATc1 modRNA for 3 days (A-E). Cell viability was measured by calcein-AM/PI staining assay. Green and red color represent living and dead cells, respectively. The percentage of living cells after NFATc1 transfection for 24 hours (F). Scale bar. 100 um. Data are mean ± SEM (n = 3). *p < 0.05 tested by ANOVA with Tukey’s multiple comparison. (TIF) [file pone.0342642.s004.tif]

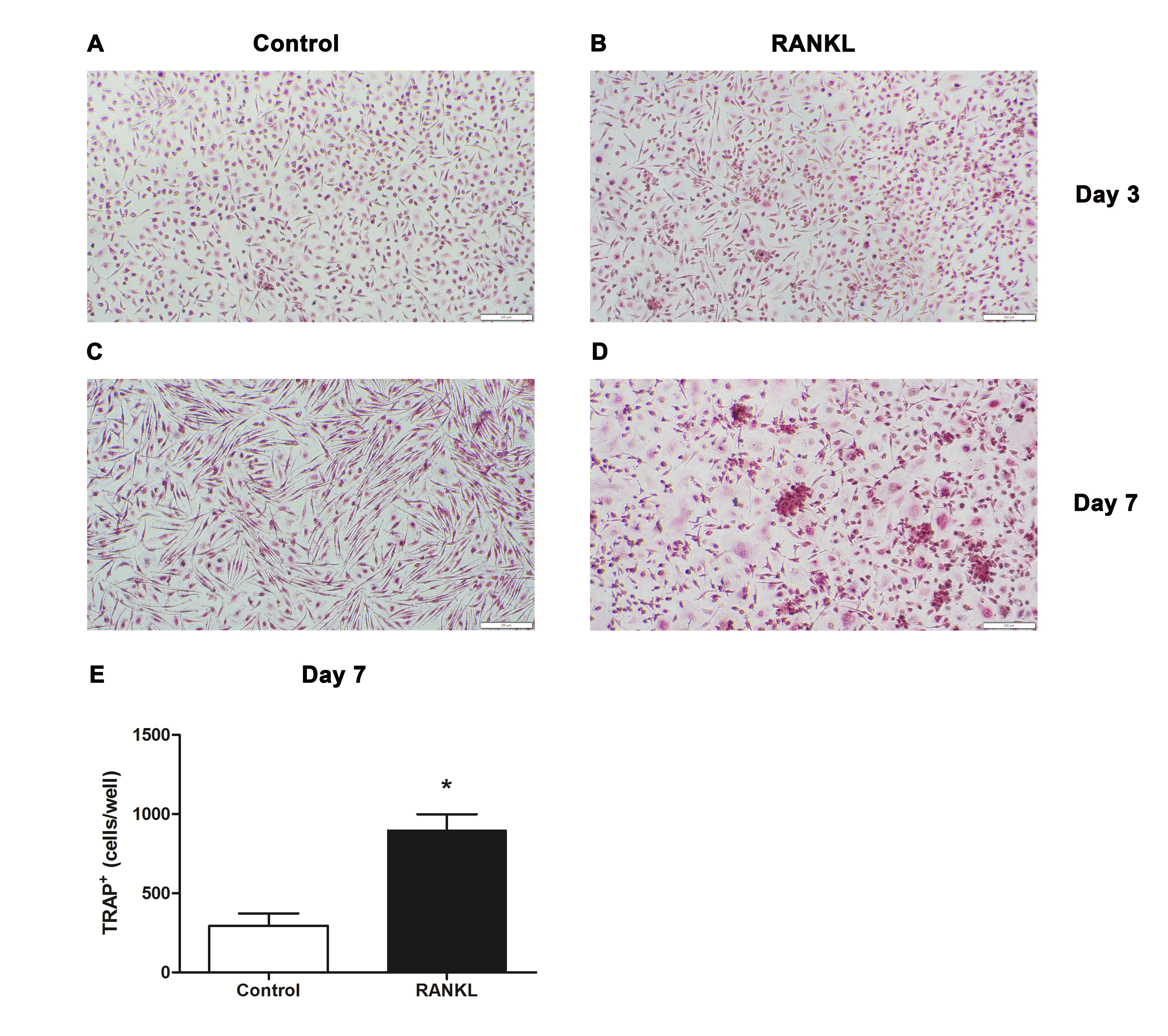

Supplement: S5 Fig — Representative images of TRAPBright cells from treated human macrophage with 25 ng/mL M-CSF (A, C) and 25 ng/mL M-CSF with 50 ng/mL RANKL (B, D) for 3 and 7 days. Scale bars = 200 µm. (E) Numbers of TRAPBright cells following treatment with M-CSF or M-CSF and RANKL for 7 days. Data are mean ± SEM (n = 3). * p < 0.05 tested by t-test. (TIF) [file pone.0342642.s005.tif]
